# Efficacy and mechanism of Shenqi Compound in inhibiting diabetic vascular calcification

**DOI:** 10.1186/s10020-023-00767-7

**Published:** 2023-12-13

**Authors:** Chan Yang, Ziyan Xie, Hanyu Liu, Xueru Wang, Zehua Zhang, Lian Du, Chunguang Xie

**Affiliations:** 1 State Key Laboratory of Biotherapy, West China Hospital, Sichuan University and Collaborative Innovation Center of Biotherapy, Chengdu, 610041 Sichuan China; 2 TCM Regulating Metabolic Diseases Key Laboratory of Sichuan Province, Hospital of Chengdu University of Traditional Chinese Medicine, Chengdu, 610075 Sichuan China; 3 Chengdu University of Traditional Chinese Medicine, Chengdu, China

**Keywords:** Shenqi Compound, Diabetic vascular calcification, Apoptosis, Extracellular matrix, Osteogenic phenotype of VSMCs, Hippo-YAP signaling pathway

## Abstract

**Background:**

Shenqi Compound (SQC) has been used in clinic for several decades in the prevention and treatment of diabetes and its complications. But this is merely a heritage of experience. The primary aim of this study is to scientifically validate the therapeutic effects of SQC on diabetic vascular calcification (DVC) in an animal model and, simultaneously, uncover its potential underlying mechanisms.

**Method:**

Spontaneous diabetic rat- Goto Kakizaki (GK) rats were selected for rat modeling. We meticulously designed three distinct groups: a control group, a model group, and an SQC treatment group to rigorously evaluate the influence of SQC. Utilizing a comprehensive approach that encompassed methods such as pathological staining, western blot analysis, qRT-PCR, and RNA sequencing, we thoroughly investigated the therapeutic advantages and the underlying mechanistic pathways associated with SQC in the treatment of DVC.

**Result:**

The findings from this investigation have unveiled the extraordinary efficacy of SQC treatment in significantly mitigating DVC. The underlying mechanisms driving this effect encompass multifaceted facets, including the restoration of aberrant glucose and lipid metabolism, the prevention of phenotypic transformation of vascular smooth muscle cells (VSMCs) into osteogenic-like states, the subsequent inhibition of cell apoptosis, the modulation of inflammation responses, the remodeling of the extracellular matrix (ECM), and the activation of the Hippo-YAP signaling pathway. Collectively, these mechanisms lead to the dissolution of deposited calcium salts, ultimately achieving the desired inhibition of DVC.

**Conclusion:**

Our study has provided compelling and robust evidence of the remarkable efficacy of SQC treatment in significantly reducing DVC. This reduction is attributed to a multifaceted interplay of mechanisms, each playing a crucial role in the observed therapeutic effects. Notably, our findings illuminate prospective directions for further research and potential clinical applications in the field of cardiovascular health.

**Graphical Abstract:**

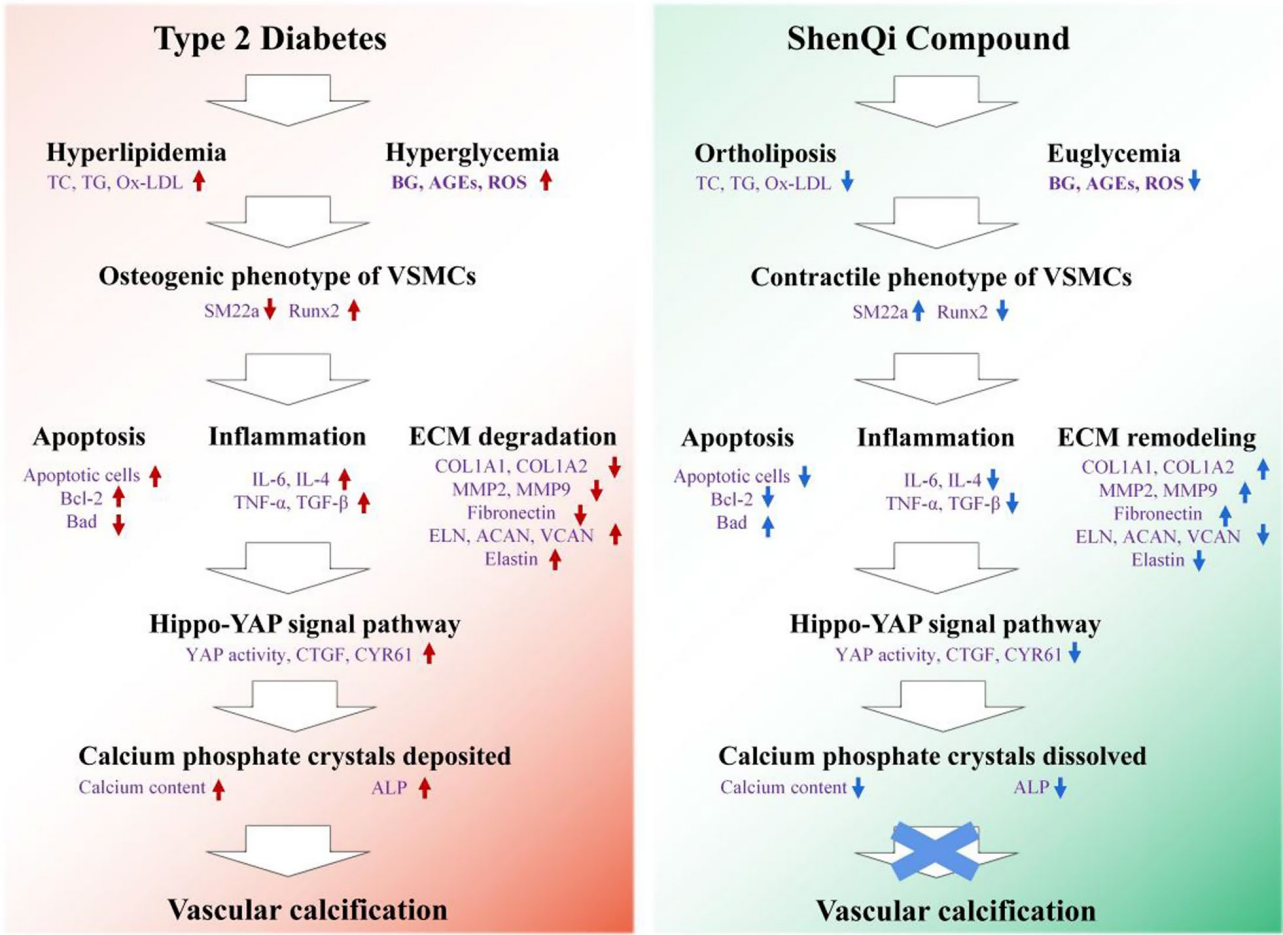

**Supplementary Information:**

The online version contains supplementary material available at 10.1186/s10020-023-00767-7.

## Highlights

- The study highlights the remarkable efficacy of SQC in significantly reducing DVC.
- The research uncovers a multifaceted interplay of mechanisms underlying the effectiveness of SQC.
- The findings shed light on potential avenues for further research and clinical applications in the realm of cardiovascular health.

**Supplementary Information:**

The online version contains supplementary material available at 10.1186/s10020-023-00767-7.

## Introduction

In recent years, the increasing prevalence of diabetes has brought about a growing concern regarding its associated complications, particularly diabetic vascular calcification (DVC) (Zheng et al. 2018; Demer and Tintut 2008). DVC, characterized by the abnormal accumulation of calcium in the arterial wall, results in arterial stiffness, impaired vascular function, and heightened cardiovascular risk (Quaglino et al. 2020). The intricate interplay between metabolic dysregulation, apoptosis, inflammation, and impaired extracellular matrix (ECM) remodeling has been identified as a pivotal factor in the development and progression of DVC (Rask-Madsen and King 2013). Consequently, there is a pressing need for innovative therapeutic strategies that can effectively target the underlying mechanisms of DVC and ameliorate its detrimental effects.

Rooted in the millennia-old Chinese herbal tradition, the Shenqi Compound (SQC) has been revered for its multifaceted therapeutic properties, which encompass anti-inflammatory, antioxidative, anti-apoptosis, and metabolic regulatory effects (Dongdong 2003; Zhang et al. 2006; Liu et al. 2019; Yang et al. 2023a). In the context of diabetes, SQC has emerged as a cornerstone in the prevention and management of diabetic macrovascular disease, which includes DVC as a critical component (Yahagi et al. 2017; Chen et al. 2020). Long-term clinical experience in China has illuminated SQC’s potential as a candidate drug for mitigating DVC (Hao et al. 2017; Wang et al. 2018a). Observations from diverse patient populations have suggested that SQC’s administration is associated with reduced vascular calcification and improved overall vascular health (Yang et al. 2023a; Fu et al. 2023). However, to translate these observations into robust therapeutic strategies, a deeper exploration of SQC’s specific effects and underlying mechanisms is indispensable.

While SQC’s reputation in Chinese traditional medicine is well-established, the scientific community recognizes the need for rigorous verification of its specific efficacy against DVC. Controlled studies, both in animal models and clinical settings, are essential to unravel the extent of SQC’s impact on vascular calcification. These investigations will shed light on whether SQC’s potential extends beyond anecdotal evidence. The scientific efficacy and potential mechanisms of SQC in countering DVC are still veiled in mystery. To fully harness its therapeutic benefits, understanding these mechanisms is paramount. Possibilities include SQC’s capacity to modulate inflammation, apoptosis, ECM remodeling, and mineral metabolism within the vascular milieu. Rigorous research can illuminate how these multifaceted actions culminate in DVC inhibition.

The integration of traditional knowledge with contemporary scientific methodologies stands as a powerful avenue for therapeutic innovation. As SQC holds historical significance in traditional Chinese medicine, substantiating its effects through modern research will not only validate its traditional usage but also foster a deeper appreciation for the potential of natural compounds in modern healthcare. Therefore, in this study, an animal model will be employed, utilizing modern bioscientific techniques, to comprehensively elucidate the efficacy and mechanisms of SQC in addressing DVC. This endeavor has the potential to revolutionize DVC management and inspire novel therapeutic strategies.

## Materials and methods

### Animals and modeling

Male GK rats, a spontaneous diabetic rat model obtained through selective inbreeding of mildly glucose-intolerant Wistar rats, and age-matched Wistar rats weighing approximately 200–250 g and aged 7 to 8 weeks, were procured from Changzhou Cavins Laboratory Animal Co., LTD. The animals were housed in a temperature-controlled room with a 12-h light/dark cycle, maintained at 22 ± 2 ℃, and kept at a relative humidity of 40–60%.

**Fig. 1:**
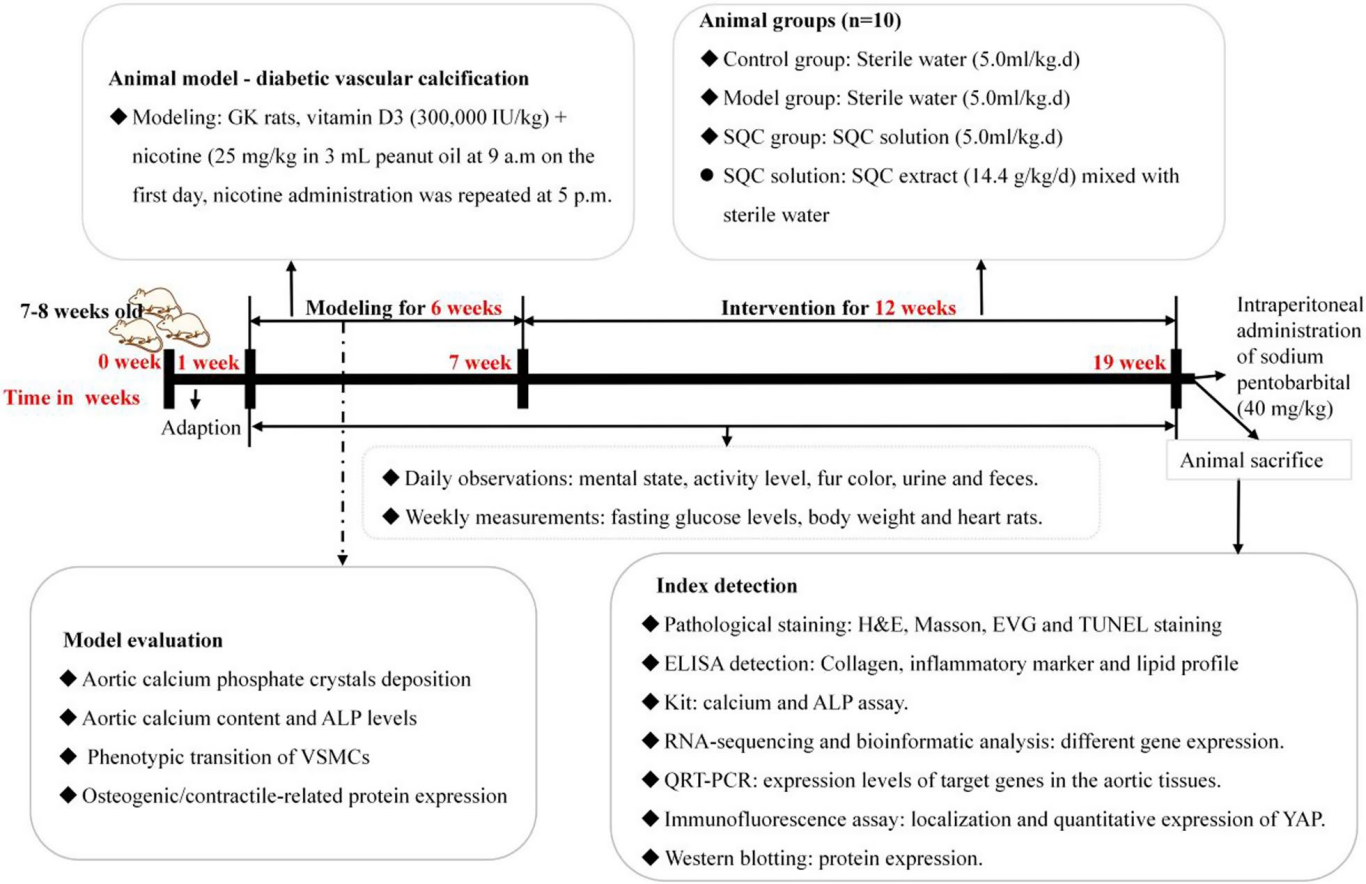
The experimental flow chart

The animal model needed for this study involved vascular calcification induced by diabetes. To eliminate vascular calcification induced by aging, we induced accelerated vascular calcification in GK rats by administering a single intraperitoneal injection of vitamin D3 (300,000 IU/kg). Simultaneously, we provided oral administration of nicotine (25 mg/kg in 3 mL peanut oil) at 9 a.m. on the first day, followed by a repeated administration of nicotine at 5 p.m. on the same day. This modeling process continued for 6 weeks, following a protocol outlined in a previous study report (Ma et al. 2021). The criteria for assessing the success of the model include the detection of aortic calcium phosphate crystal deposition, aortic calcium content, ALP levels, phenotypic transition of VSMCs, and the expression of osteogenic and contractile-related proteins. Prior to the formal experiment, we conducted multiple pilot experiments, and the results were consistent with the descriptions in the existing literature (Ma et al. 2021), confirming the successful establishment of the vascular calcification model. After the modeling period, all GK rats were randomly divided into Model Group (intragastric administration of saline, 5.0 ml/kg/day) and SQC Group (intragastric administration of medium-dose SQC extract, 14.4 g/kg/day). Wistar rats were assigned to the Control Group. Each group consisted of 10 rats. The Control group received the same volume of saline injections (5.0 ml/kg/day). There were no significant differences in body weight among the groups prior to treatment. After 12 weeks of treatment, all rats were humanely euthanized via intraperitoneal injection of sodium pentobarbital (40 mg/kg). The detailed timeline is shown in Fig. 1.

The utmost priority was given to ensuring the well-being of the animals and minimizing any potential distress. All animal care and experimental procedures strictly adhered to the Animal Management Rule of the Ministry of Health of the People’s Republic of China, as well as the guidelines outlined in the ‘Care and Use of Laboratory Animals’ by the United States National Institutes of Health. Additionally, the study protocols obtained approval from the Ethics Committee of the Hospital of Chengdu University of Traditional Chinese Medicine (approval number: 2021DL008).

### Antibodies and reagents

Radio-Immunoprecipitation Assay (RIPA, 89901) and FBS (10099-141) were purchased from Thermo Fisher Scientific Co., Inc. BSA, SDS, and 1-Bromo-3-chloropropane (BCP) were purchased from Sigma-Aldrich. Metformin (H20023371) was purchased from Sino-us Shanghai Squibb Pharmaceutical Co., LTD. Antibodies for RUNX2 (ab76956), BMP2 (ab284387), and SM22α (ab14106) were purchased from Abcam. The antibody for GAPDH (Cat No. 60004-1-Ig) was purchased from Proteintech. Antibodies for Mouse IgG, HRP-linked antibody (7076), and Rabbit IgG, HRP-linked antibody (7074) was purchased from CST.

### Alizarin Red S and Von Kossa staining

For Alizarin Red S staining, paraffin-embedded sections were dehydrated, incubated with Alizarin Red S solution to bind with calcium deposits, washed, dehydrated again, and then cleared before mounting on glass slides. In the case of Von Kossa staining, tissue sections underwent deparaffinization and rehydration, followed by incubation with silver nitrate solution and exposure to UV light, counterstaining, dehydration, clearing, and finally, mounting on glass slides (Additional file 1: Sect. “Materials and methods”). These staining techniques allowed the visualization of calcium deposits within the tissue sections, enabling examination under a light microscope for the observation of distinct staining patterns in calcium-rich areas.

### Quantification of calcium deposition

The calcium content in the arteries was quantified using a commercially available Ca assay kit (Jiancheng, Nanjing, China), following the provided instructions. Arteries were prepared by sonication in deionized water, and after centrifugation, 10% of the supernatant was collected for measurement. Calcium content was determined by mixing the supernatant with Methyl thymol blue (MTB), an Alkaline solution, and a Protein clarifier, and co-incubating for 5 min at room temperature. Absorbance at 610 nm was measured using a microplate reader. Protein concentration was determined with the Bradford protein assay, and calcium content was normalized to protein levels, providing precise quantification of calcium deposition in the arteries while accounting for tissue protein variations.

### H&E staining and histopathology

Following treatment, thoracic aortas, heart, liver, kidney, and lungs were dissected, fixed in 4% paraformaldehyde, and embedded in paraffin for thin sectioning (5–7 μm). H&E staining involved deparaffinization, rehydration, and nuclear-cytoplasmic visualization. A blinded pathologist assessed structural integrity, vascular wall architecture, cell morphology, and histopathological changes under a light microscope, systematically examining multiple sections from each group. Findings were documented with captured images.

### Masson and Verhoeff’s Van Gieson (EVG) staining

The process involves deparaffinization and rehydration of the tissue, Masson staining to visualize nuclei, cytoplasm, and collagen fibers, and EVG staining to enhance the visibility of elastic fibers and connective tissue components. Subsequently, the stained sections are dehydrated, cleared, and mounted on slides for examination under a light microscope, revealing distinct coloration patterns for the observation of tissue components (Additional file 1: Sect. "Results").

### Quantification of Collagen levels detection

Collagens were quantified in this study using an ELISA. Pancreatic tissue homogenates were prepared and aliquoted. A 96-well microtiter plate was coated with anti-collagen antibodies overnight, followed by blocking with a blocking buffer. Standards and samples were added to the plate, and after incubation, any unbound analytes were washed away. Subsequently, a detection antibody specific to collagen, which was conjugated with an enzyme, was added, and the plate was washed again. A substrate solution was then added to initiate a colorimetric reaction, and after a defined incubation period, the reaction was stopped. The absorbance of the resulting color in each well was measured using a microplate reader at an appropriate wavelength.

### TUNEL staining

Collagens were quantified in this study using an ELISA. Pancreatic tissue homogenates were prepared and aliquoted. A 96-well microtiter plate was coated with anti-collagen antibodies overnight, followed by blocking with a blocking buffer. Standards and samples were added to the plate, and after incubation, any unbound analytes were washed away. Subsequently, a detection antibody specific to collagen, which was conjugated with an enzyme, was added, and the plate was washed again. A substrate solution was then added to initiate a colorimetric reaction, and after a defined incubation period, the reaction was stopped. The absorbance of the resulting color in each well was measured using a microplate reader at an appropriate wavelength.

### RNA-sequencing and bioinformatic analysis

We performed RNA-sequencing (RNA-seq) to analyze aortic tissue transcriptomes, examining gene expression changes related to diabetic aortic calcification, apoptosis, and the effects of SQC treatment. Total RNA was extracted from aortic tissues using a commercial kit and assessed for quality and quantity. Library preparation included mRNA purification, fragmentation, cDNA synthesis, adapter addition, and PCR amplification. Library quality was evaluated before sequencing on an Illumina platform, which generated paired-end reads. Raw data were preprocessed to remove artifacts. Clean reads were aligned to the reference genome using STAR, and differential gene expression was analyzed with DESeq2. Functional enrichment analysis identified relevant GO terms and KEGG pathways. Sequencing data were deposited in the NCBI Sequence Read Archive, and bioinformatic analyses were conducted using R (version 3.6.2, https://www.r-project.org/).

### Flow cytometry analysis of VSMCs apoptosis

Preparation of VSMCs Suspensions: Arterial tissue, typically from the aorta, was dissected from sacrificed rats and carefully separated from the adventitia. The tunica media, primarily comprising VSMCs, was minced into small pieces. Enzymatic digestion was performed by incubating the tissue in an enzyme solution. The resulting cell suspension was filtered to remove undigested tissue fragments, and VSMCs were pelleted by centrifugation (Additional file 1: Section 3). After assessing viability and concentration, the VSMC suspension was prepared for subsequent experiments.

Annexin V-FITC and Propidium Iodide Staining: Centrifuged the VSMC suspension at 300 × g for 5 min, then resuspend the pellet in cold PBS and repeat the centrifugation step. After resuspending the pellet in binding buffer at a concentration of 1 × 10^6^ cells/ml, transfer 100 μl of the VSMC suspension to a flow cytometry tube. Add 5 μl of Annexin V-FITC, gently mix, and incubate for 15 min. Follow this with the addition of 10 μl of PI solution, another gentle mix, and a 5-min incubation in the dark. Adjust each sample’s volume to 500 μl with binding buffer. Analyze the samples using a flow cytometer, ensuring appropriate instrument settings and gating. Collect data from a minimum of 10,000 events per sample for subsequent analysis (Additional file 1: Section 4).

### ELISA detection of inflammatory and lipid metabolism marker

Rat aortic tissue samples were collected and homogenized to ensure uniformity. We then used an ELISA kit following standard instructions: we added the homogenates to designated wells, incubated them, and washed away any unbound substances. A detection antibody, conjugated with an enzyme, specifically bound to captured analytes. A chromogenic substrate was added to induce a proportional color change. After measurement with a microplate reader, we determined analyte concentrations by comparing absorbance readings to a standard curve.

### Immunofluorescence (IF) assay

We conducted an IF assay to assess YAP protein expression and localization in aortic tissues. Paraffin-embedded sections were prepared, underwent antigen retrieval in a Tris/EDTA solution (pH 9.0), and were then permeabilized using Triton X-100 for enhanced antibody penetration. Anti-YAP antibodies were applied and left to bind to their epitopes overnight at 4 °C. After washing away unbound primary antibodies, fluorescent secondary antibodies were added, selected to match the species. Following another wash, DAPI staining visualized cell nuclei. Sections were mounted with an anti-fade medium for fluorescence preservation. Fluorescence microscopy captured the signals with appropriate filters. Control experiments without primary antibodies were conducted to confirm signal specificity. Quantification of immunofluorescence signals was performed using Image-Pro Plus software (version 6.0), and statistical analysis compared signals across different groups.

### Quantitative real-time polymerase chain reaction (qRT-PCR)

Total RNA was extracted using a Thermo Fisher Scientific RNA extraction kit, following the manufacturer’s instructions. The extracted RNA was quantified and assessed for quality with a spectrophotometer. Subsequently, cDNA was synthesized using a reverse transcription kit, converting RNA into cDNA with reverse transcriptase enzyme and oligo(dT) primers. The resulting cDNA served as the template for qRT-PCR, with specific primer pairs designed and synthesized for target genes. qRT-PCR reactions were set up with SYBR Green-based master mix and designed primers, and amplification was performed in a real-time PCR instrument. The housekeeping gene GAPDH was used for gene expression normalization. The comparative threshold cycle (Ct) method was applied to calculate relative gene expression levels. Statistical analysis was conducted to determine intergroup differences in gene expression. Primer sequences used in the analysis are provided in Additional file 2: Table S1.

### Western blotting analysis

Protein extraction involved a lysis buffer with protease and phosphatase inhibitors. After quantification with a Bradford assay, equal protein amounts underwent SDS-PAGE by molecular weight. Proteins were transferred to nitrocellulose or PVDF membranes using a transfer system. Successful transfer was confirmed with Ponceau S staining or molecular weight markers. Membranes were blocked with 5% non-fat milk and then incubated overnight at 4 °C with specific primary antibodies. After washing, secondary antibodies with HRP or fluorophores were used for detection. Chemiluminescent signals were captured via imaging systems, and fluorescent signals were detected with a fluorescence system. Protein levels were quantified and normalized to GAPDH using Image J.

### Statistical analysis

Quantitative data were presented as the mean and standard deviation (mean ± SD) from a minimum of three independent experiments. All statistical analyses were conducted using SPSS statistical software program version 20.0. For comparisons between two groups, Student’s t-tests were applied, and for comparisons involving multiple groups, a one-way analysis of variance (ANOVA) test was used, followed by post hoc comparisons using Tukey’s multiple comparisons test. A significance level of *P* < *0.05* was considered statistically significant.

## Results

### Determination of SQC Dosage

To validate the efficacy and safety of SQC, we initially established three different dosage groups: low (7.2 g/kg/d), medium (14.4 g/kg/d), and high (28.8 g/kg/d), following our previous literature (Yang et al. 2023b). To validate the effectiveness of the experimental intervention, we concurrently included metformin, a first-line medication for diabetes, as the positive control group in our study. The results shown in Additional file 7: Figure S1A, it demonstrated a significant decrease in blood glucose levels within the metformin group, confirming the efficacy of the experimental intervention. Additionally, both the medium and high dosage groups of SQC exhibited a substantial reduction in blood glucose levels. While the low dosage group also exhibited a glucose-lowering effect, its efficacy was inconsistent, with fluctuating effects. A concern arose as the high dosage group of SQC demonstrated a trend of decreased body weight in rats compared to the other groups, while there was no significant difference in body weight between the low and medium dosage groups of SQC and the other groups (Additional file 7: Figure S1B). This raised concerns about potential side effects due to the high dosage of SQC. Therefore, we conducted further assessments of the safety of SQC in rats. The detail shown in Additional file 1: Section 5. As SQC is intended for clinical use, we limited our evaluation to serum biochemical marker analysis and H&E staining of major rat organs at the current dosage levels. Serum levels of aspartate transaminase (AST), alanine transaminase (ALT), Cre, and creatine kinase MB (CKMB) are commonly used indicators to assess drug-induced organ toxicity, with AST and ALT evaluating liver toxicity, Cre estimating renal toxicity, and CKMB assessing cardiac toxicity (Additional file 1: Sect.  6). The results of the serum biochemical tests showed no significant differences in AST, ALT, Cre and CKMB levels in rats administered low and medium doses of SQC compared to the normal control (NC) group (*P* > *0.05*, Additional file 8: Figure S2A), indicating no apparent liver, kidney or heart toxicity at these dosage levels. Furthermore, observing the morphological changes in vital organs of the rats (Additional file 1: section 7). The H&E staining results (Additional file 8: Figure S2B) also revealed no significant damage to major organs such as the heart, liver, spleen, kidney, and lungs when rats were administered SQC at low and medium doses. However, a disappointing finding was that when rats were administered high doses of SQC, there was a tendency for increased AST and ALT levels compared to the NC group, suggesting a potential impact on liver function at this high dosage (Additional file 8: Figure S2A). Nonetheless, as a precautionary measure, we chose to use the medium dosage (14.4 g/kg/d) group of SQC for subsequent experiments in the interest of safety.

### SQC alleviation of DVC

**Fig. 2:**
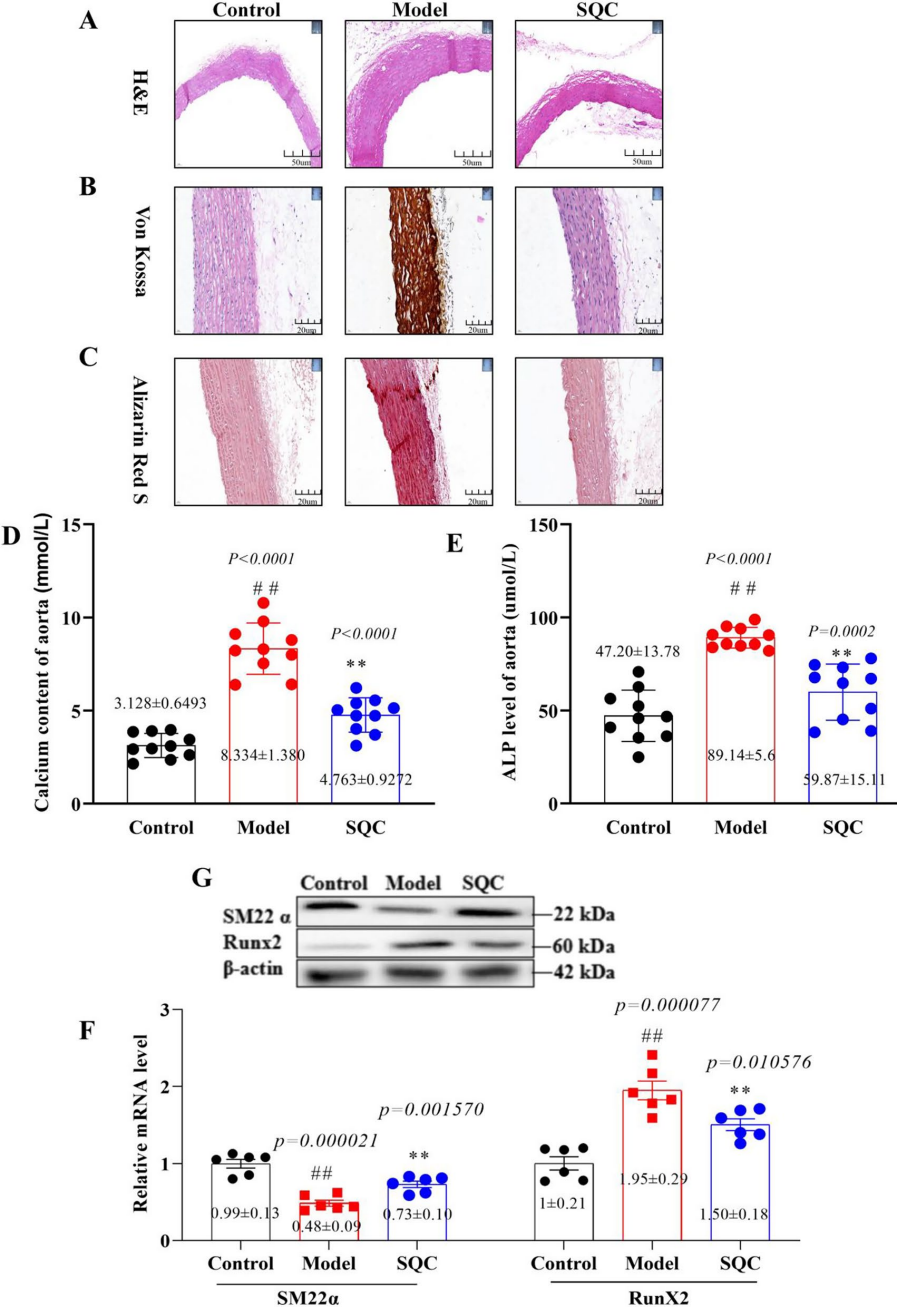
SQC alleviation of aortic calcification. **A** Representative HE staining (scale bar, 200 μm) (n = 4). **B** Representative Von Kossa staining (scale bar, 200 μm) (n = 4). **C** Representative Alizarin Red S staining (scale bar, 200 μm) (n = 4). **D** The levels of calcium content in rat aortas. **E** The levels of ALP content in rat aortas. **F** The relative mRNA levels of SM22a and RUNX2. **G** The protein levels of SM22a and RUNX2. Model group compared with control group: ^*#*^*P* ≤ *0.05*, ^*##*^*P* ≤ *0.01*. SQC groups compared with model group: *P ≤ 0.05, **P ≤ *0.01*

To demonstrate the impact of SQC on DVC, we began by visually assessing the external morphology of the aortas in each group. No significant changes were discernible to the naked eye (Additional file 8: Figure S2C). Subsequently, we employed H&E, Von Kossa, and Alizarin Red staining techniques to investigate the therapeutic impact of SQC on pathological morphological changes and aortic calcium deposition. Microscopic examination revealed substantial histological alterations in the aortas of the model group, including notable endothelial cell loss, increased vascular wall thickness, cell swelling, and disordered cell arrangement. However, in the SQC group, these abnormal changes were ameliorated (Fig. 2A). Furthermore, the model group rats exhibited significant calcium deposition in their aortas compared to the control group (Von Kossa staining exhibited a significant brown coloration, while Alizarin Red S staining displayed a pronounced deep red coloration). After treatment with SQC, the abnormal changes in the aortas of the treatment group rats significantly improved (Fig. 2B and C). These tissue-level improvements indicate the inhibitory effect of SQC on DVC. Furthermore, measurements of calcium and alkaline phosphatase (ALP) content in the rat thoracic aorta tissues indicated that SQC treatment significantly reduced calcium and ALP levels compared to the model group (*P* < *0.05*, Fig. 2D and E), providing further confirmation of SQC’s inhibitory effect on DVC. Moreover, the phenotypic transition of VSMCs from a contractile phenotype to an osteogenic phenotype is a characteristic feature of DVC (Shi et al. 2020). Consequently, qRT-PCR and Western blot results exhibited a significant decrease in the expression of the osteogenic-related protein, Runx2, and a significant increase in the expression of the contractile-related protein, SM22α, in the SQC group when compared to the model group (*P* < *0.05*, Fig. 2F and G), suggesting SQC’s inhibitory effect on DVC at both the molecular and protein levels. In summary, these findings underscore SQC’s inhibitory effect on aortic calcification in diabetic rats.

### SQC inhibition of glycolipid toxicity

**Fig. 3:**
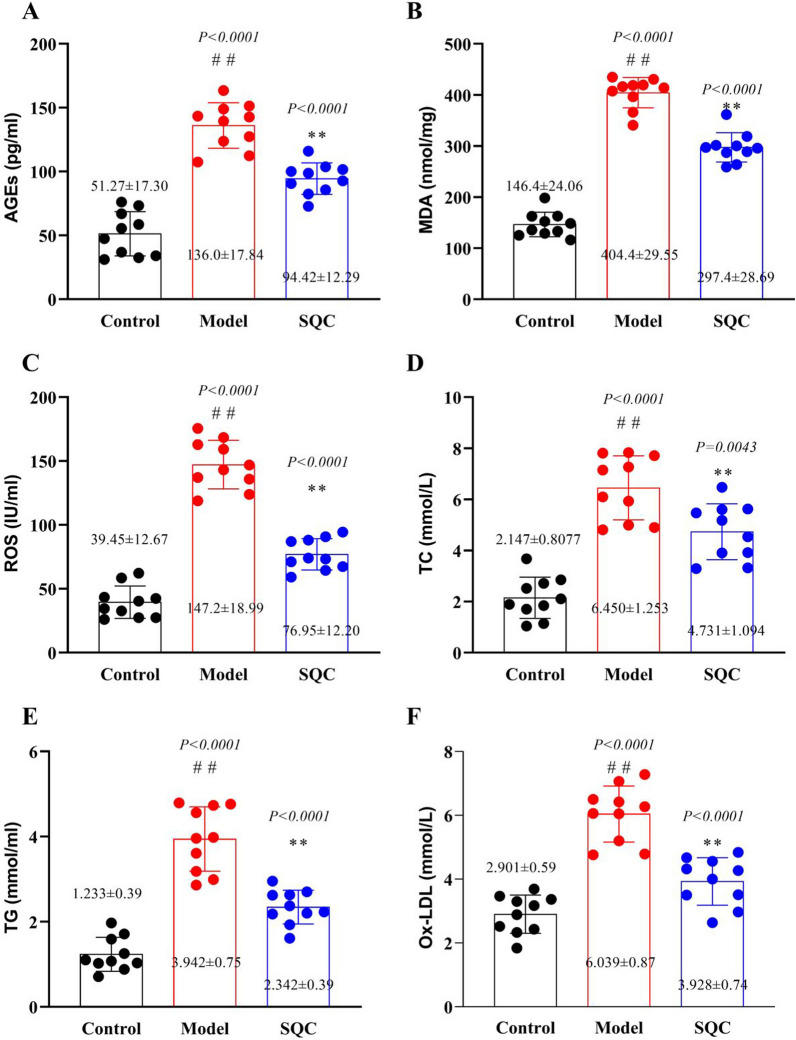
SQC Inhibition of Glycolipid toxicity. **A** AGEs levels. **B** MDA levels. **C** ROS levels. **D** TC levels. **E** TG levels. **F** Ox-LDL levels. Model group compared with control group: ^*#*^*P* ≤ *0.05*, ^*##*^*P* ≤ *0.01*. SQC groups compared with model group: *P ≤ 0.05, **P ≤ *0.01*

An increasing body of research demonstrates that in the realm of cardiovascular protection, multi-target natural medicines, particularly Chinese herbs or compounds, offer unparalleled advantages when compared to single-target pharmaceuticals (Hao et al. 2017; Wang et al. 2018a; Won et al. 2017). Consequently, we embarked on a more comprehensive investigation into the potential mechanisms through which SQC counters DVC. Through a thorough review of the literature, it became evident that in the context of diabetes-induced hyperglycemia, the prolific generation of advance glycosylation end products (AGEs) and reactive oxygen species (ROS) initiates oxidative stress, serving as the catalyst for DVC (Li et al. 2022; Ghosh et al. 2020). Our study results demonstrate that, following SQC treatment, there is a remarkable reduction in AGEs, malondialdehyde (MDA), and ROS levels when compared to the model group (*P* < *0.05*, Fig. 3A–C). These findings strongly suggest that SQC effectively curtails glycotoxicity. Furthermore, the literature confirms that under high-fat conditions, lipotoxicity is a critical trigger for DVC (Ma et al. 2021; Ghosh et al. 2020). As shown in Fig. 3D–F, the results indicate that after SQC treatment, SQC significantly lowers rat total cholesterol (TC), triglyceride (TG), and oxidized low-density lipoprotein (Ox-LDL) levels (*P* < *0.05*), signifying its substantial inhibitory effect on lipotoxicity. Taken together, our findings strongly suggest that SQC may effectively impede the occurrence and progression of DVC by addressing both glycotoxicity and lipotoxicity at their roots in diabetic rats.

### SQC inhibition of apoptosis

**Fig. 4:**
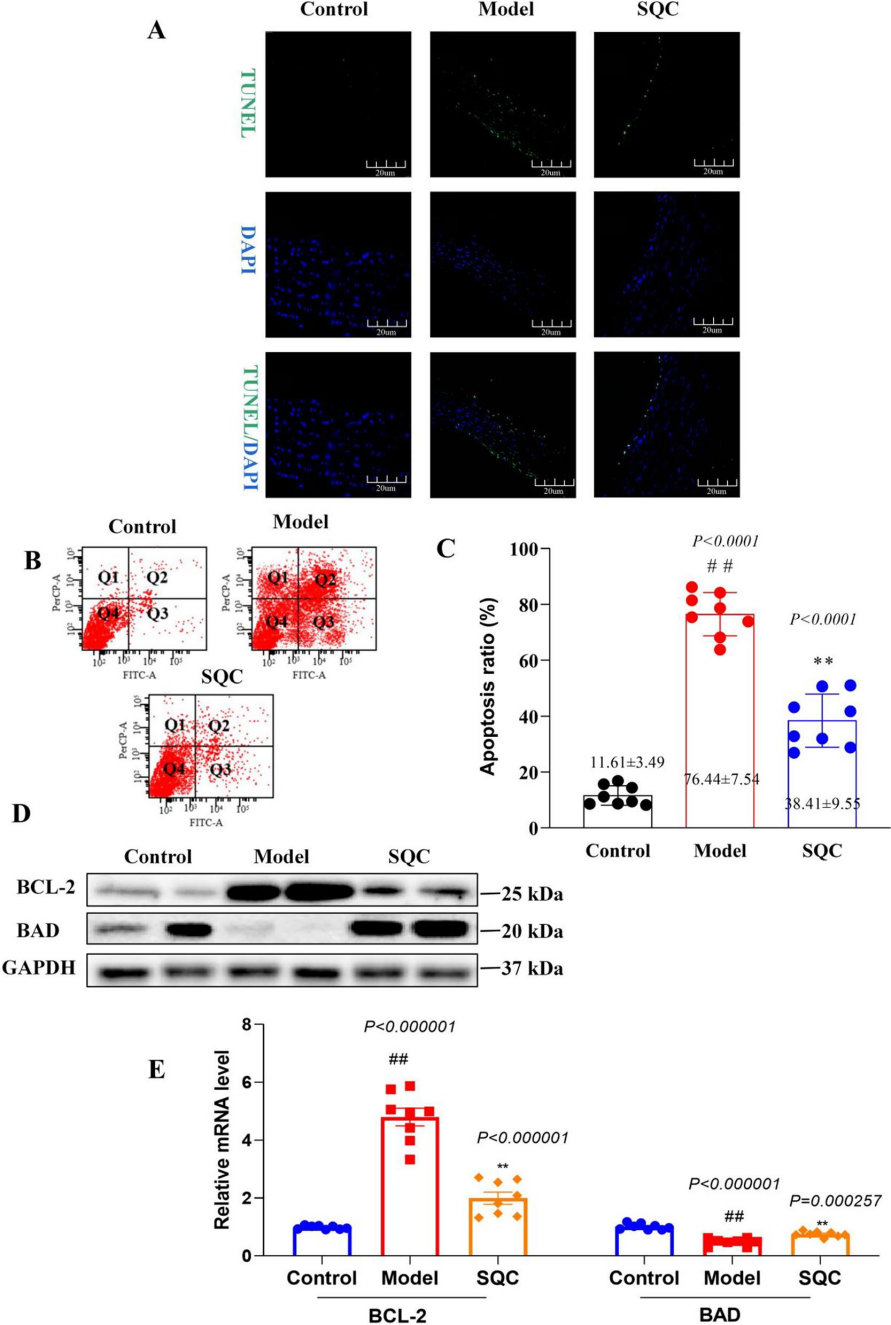
SQC Inhibition of Apoptosis. **A** Representative TUNEL staining (scale bar, 200 μm) (n = 4). **B** Representative flow cytometry analysis (scale bar, 200 μm) (n = 4). **C** The apoptosis rats of aortic cell. **D** The protein levels of Bad and Bcl-2. **E** The relative mRNAs levels of Bad and Bcl-2. Model group compared with control group: ^*#*^*P* ≤ *0.05*, ^*##*^*P* ≤ *0.01*. SQC group compared with model group: *P ≤ 0.05, **P ≤ *0.01*

Existing literature highlights that cell apoptosis is one of the most important processes in vascular calcification (Ghosh et al. 2020; Wang et al. 2020). Upon microscopic observation through TUNEL staining, it was evident that after SQC treatment, apoptosis of aortic wall cells in diabetic rats was significantly inhibited (Fig. 4A). Furthermore, after isolating VSMC cells from the aortic wall tissue of the rats and subjecting them to flow cytometry analysis, it was confirmed that SQC can indeed suppress apoptosis in aortic wall VSMCs (Fig. 4B and C). Additionally, qRT-PCR and Western blot analysis demonstrated a notable restoration of aberrantly altered apoptotic markers, including Bad and Bcl-2, in the model group following SQC treatment (*P* < *0.05*, Fig. 4D and E). The administration of SQC exhibited a protective effect against aortic apoptosis, resulting in a significant reduction in apoptotic events within the aortic tissues. This observation highlights the inhibitory role of SQC in the occurrence and progression of aortic apoptosis.

### SQC inhibition of inflammatory factor release

**Fig. 5:**
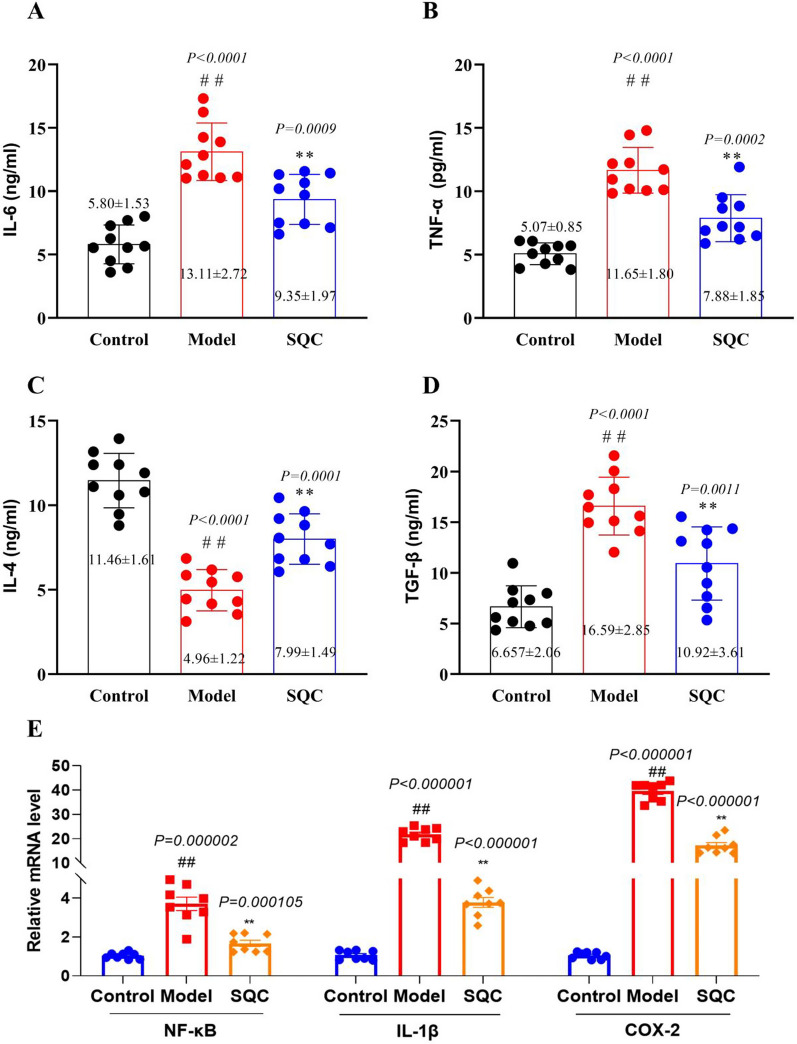
SQC inhibition of inflammatory factor release. **A** The level of IL-6. **B** The level of TNF-α. **C** The level of IL-4. **D** The level of TGF-β. **E** The relative mRNA level of key inflammatory markers. Model group compared with control group: ^*#*^*P* ≤ *0.05*, ^*##*^*P* ≤ *0.01*. SQC group compared with model group: *P ≤ 0.05, **P ≤ *0.01*

Various pro-inflammatory factors such as interleukin 6 (IL-6), interleukin 4 (IL-4), tumor necrosis factor-α (TNF-α), transforming growth factor-β (TGF-β), and Nuclear Factor Kappa Beta (NF-KB), interleukin-1β (IL-1β), and cyclooxygenase-2 (COX-2) are released within the vascular wall, accelerating the formation of vascular calcification in diabetic rats (Ghosh et al. 2020; Lee et al. 2020). Consequently, we delved further into investigating the impact of SQC on these inflammatory factors. By employing ELISA to analyze inflammatory factors within the rat aortic tissues, our findings revealed that, following SQC treatment, there was a significant reduction in the levels of IL-6, IL-4, TNF-α, and TGF-β (*P* < *0.05*, Fig. 5A–D). Furthermore, qRT-PCR analysis demonstrated a notable restoration of aberrantly altered key inflammatory markers, including NF-KB, IL-1β, and COX-2 in the model group following SQC treatment (*P* < *0.05,* Fig. 4E). These results suggest that the inhibitory effect of SQC on DVC might be linked to its ability to suppress inflammation.

### SQC remodeling of ECM

**Fig. 6:**
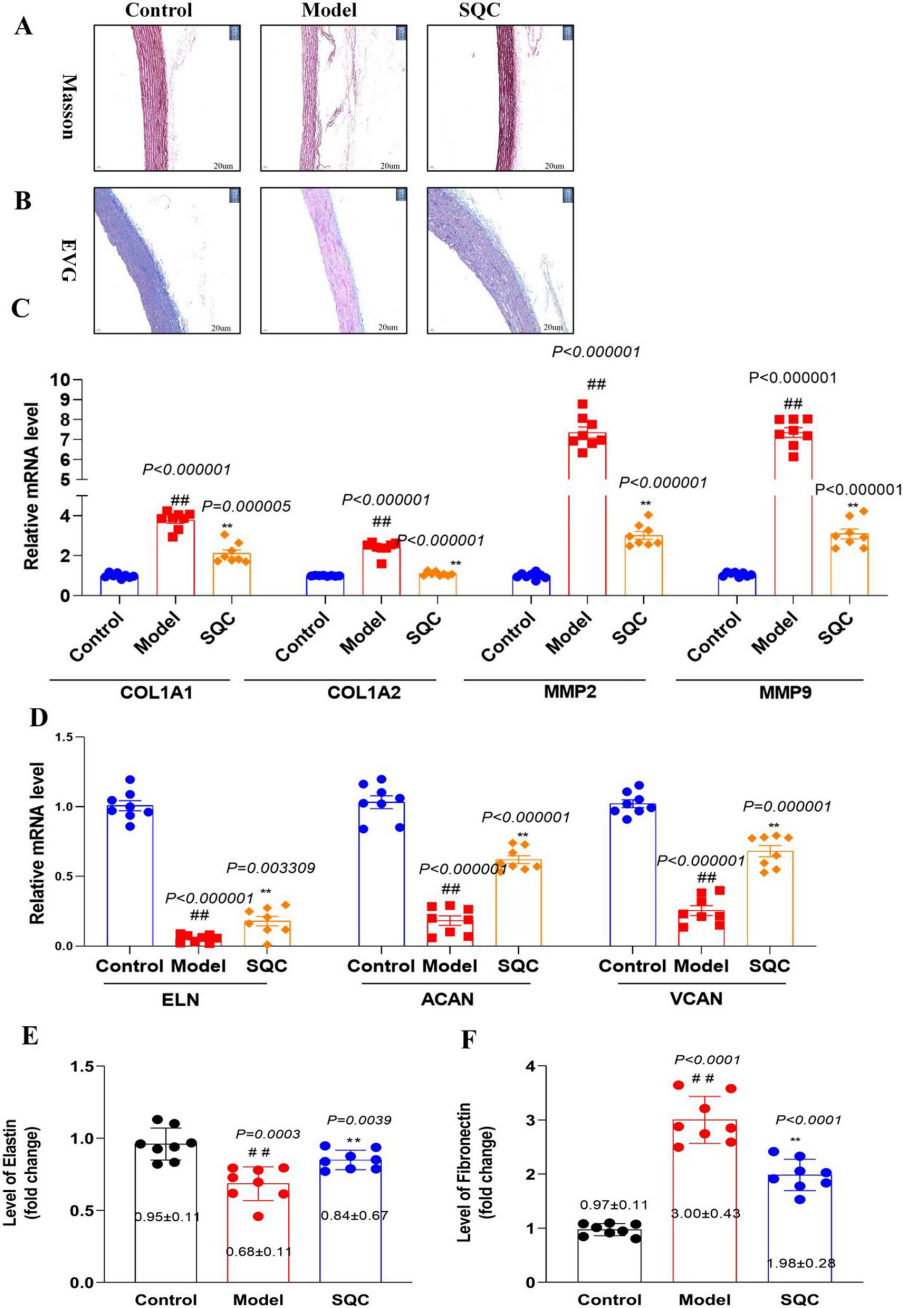
SQC remodeling of ECM. **A** Representative Masson staining (scale bar, 200 μm) (n = 4). **B** Representative VEG staining (scale bar, 200 μm) (n = 4). **C** The downregulated relative mRNAs levels of ECM synthesis, degradation and remodeling. **D** The upregulated relative mRNAs levels of ECM synthesis, degradation and remodeling. **E** The levels of Elastin. **F** The levels of Fibronectin. Model group compared with control group: ^*#*^*P* ≤ *0.05*, ^*##*^*P* ≤ *0.01*. SQC group compared with model group: *P ≤ 0.05, **P ≤ *0.01*

The degradation of the ECM creates a microenvironment conducive to the deposition of calcium phosphate crystals, which forms the pathological basis of vascular calcification (Ghosh et al. 2020; Lee et al. 2020). As a result, the remodeling of the ECM emerges as a pivotal pathway for suppressing DVC. A range of methodologies and parameters can be employed to evaluate ECM remodeling. To initiate this assessment, we utilize Masson’s Trichrome and EVG staining to visually examine and analyze variations in collagen fiber density, organization, and distribution. Masson staining vividly portrays the typical distribution of collagen fibers, manifesting as a shade of blue in the control group. In contrast, the model group exhibits a noteworthy increase in collagen fiber deposition, leading to an intensified blue staining pattern. Subsequent SQC treatment brings about a visible reversal of abnormal collagen fiber deposition, progressively restoring a more typical appearance (Fig. 6A). Similarly, EVG staining highlights a well-structured and uniform distribution of elastic fibers across the tissues. Elastic fibers typically exhibit a black or dark brown appearance due to their affinity for the staining components within control group. Conversely, the model group shows apparent thinning, fragmentation, or disarray of elastic fibers in affected blood vessels, contributing to weakened vessel walls. Following SQC treatment, these damaged elastic fibers gradually regain their normal appearance (Fig. 6B). In addition, we conducted qRT-PCR analyses of several key genes associated with ECM synthesis, including collagen type I alpha 1 (COL1A1), collagen type I alpha 2 (COL1A2), and elastin (ELN), ECM degradation, such as matrix metalloproteinase (MMP) 2 and MMP9, and ECM remodeling, encompassing aggrecan (ACAN) and versican (VCAN). Our findings indicate that, in comparison to the model group, COL1A1, COL1A2, MMP2, and MMP9 were significantly downregulated in the SQC group (*p* < *0.05*, Fig. 6C), while ELN, ACAN, and VCAN displayed a significant upregulation (*P* < *0.05*, Fig. 6D). Furthermore, ELISA assays revealed a reduction in Elastin levels within the model group when compared with the control group (*P* < *0.05,* Fig. 6E). SQC treatment, on the other hand, led to a substantial restoration of Elastin levels (*P* < *0.05*, see Fig. 6E). Notably, Fibronectin, a vital component of the ECM playing a pivotal role in cell adhesion and tissue repair, exhibited significantly lower levels in the SQC group compared to the model group (*P* < *0.05*, see Fig. 6F).

### SQC modulation of Hippo-YAP pathway

**Fig. 7:**
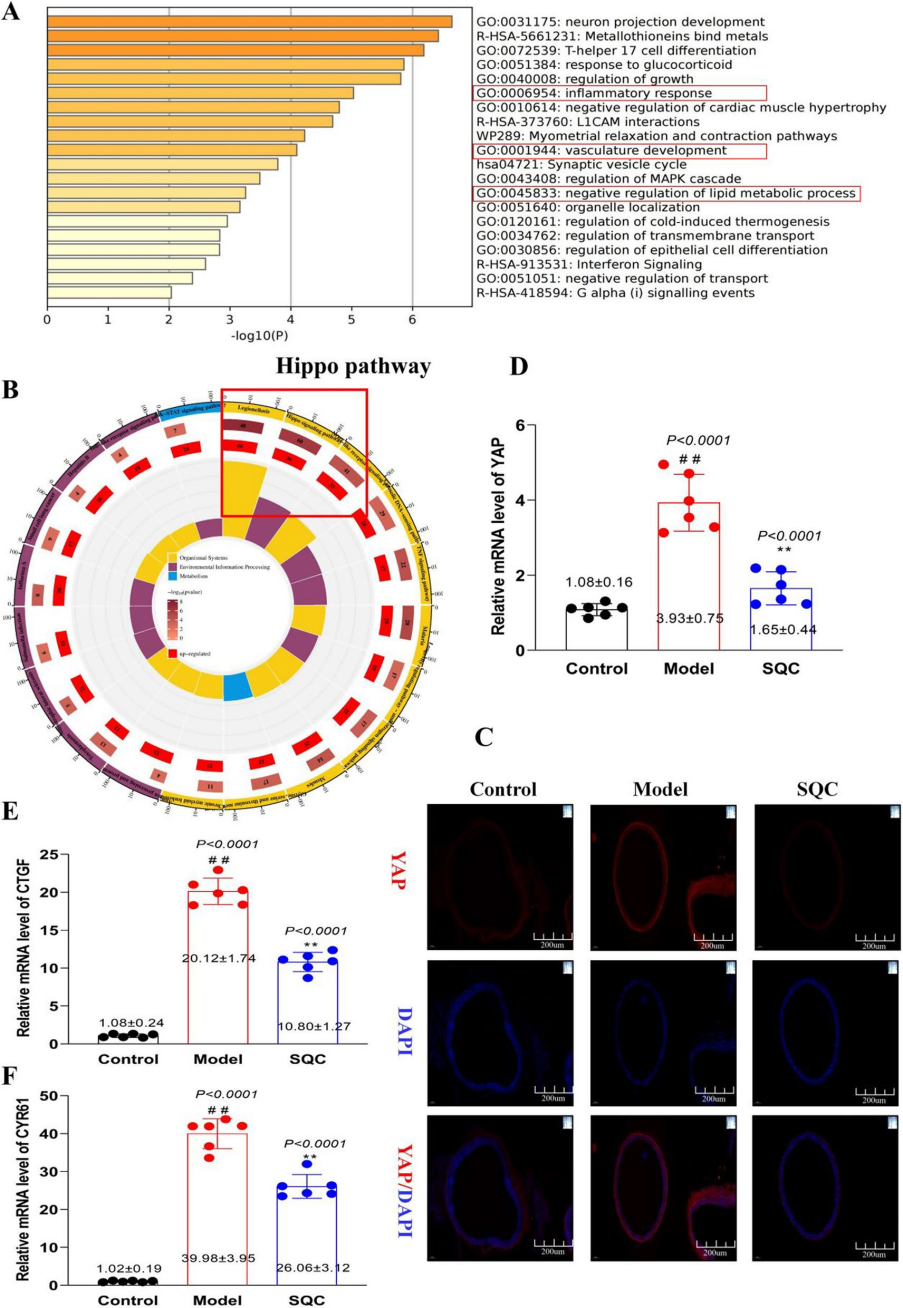
SQC activation of Hippo-YAP pathway. **A** The GO enrichment analysis between SQC and model group. **B** The KEGG enrichment analysis between SQC and model group. **C** Representative IF staining (scale bar, 200 μm) (n = 4). **D** The relative mRNA level of YAP, CTGF and CYR61. Model group compared with control group: ^*#*^*P* ≤ *0.05*, ^*##*^*P* ≤ *0.01*. SQC group compared with model group: *P ≤ 0.05, **P ≤ *0.01*

Through a comprehensive exploration at both the tissue and cellular levels, it becomes evident that the mechanisms through which SQC inhibits DVC encompass the suppression of glycotoxicity and the prevention of VSMC phenotypic osteogenic transition. These actions, on one hand, curtail cell apoptosis and inflammatory responses, and on the other hand, reshape the ECM, ultimately thwarting the deposition of calcium phosphate crystals. To further substantiate this conclusion, we conducted RNA sequencing to delve into the potential molecular mechanisms by which SQC inhibits VC. The analysis revealed significant alterations in the transcriptome profiles of aortic tissues following SQC treatment. In the model group compared to the control group, we identified a total of 1005 differentially expressed genes (DEGs), with 516 upregulated and 489 downregulated (Additional file 3: Table S2). The SQC group compared to the model group exhibited 40 DEGs, consisting of 35 upregulated and 5 downregulated genes (Additional file 4: Table S3). A functional enrichment analysis of the DEGs was performed to gain a deeper understanding of the biological processes and pathways influenced by SQC intervention. The GO analysis revealed that the DEGs commonly regulated in the SQC group were consistent with the previously mentioned processes, including the inflammatory response, vasculature development, and lipid metabolic processes (Fig. 7A and Additional file 5: Table S4). Moreover, the KEGG pathway analysis revealed a significant enrichment of DEGs in the Hippo pathway in response to SQC treatment (Fig. 7B and Additional file 6: Table S5). This finding further highlights the potential involvement of the Hippo signaling pathway in mediating the inhibitory effects of SQC on DVC. As depicted in Additional file 9: Figure S3, the Yes-associated protein (YAP) plays a crucial role in the Hippo signaling pathway. Our results revealed that SQC administration led to the activation of the Hippo-YAP pathway, as supported by several key observations. Firstly, IF staining demonstrated a decrease in the nuclear translocation of YAP in the SQC group compared to the model group (Fig. 7C). This reduction in nuclear translocation indicates the inhibition of YAP activity and nuclear accumulation, which is associated with its transcriptional activity and downstream effects (Totaro et al. 2018). Furthermore, qRT-PCR analysis revealed decreased mRNA expression levels of YAP and its target genes, such as connective tissue growth factor (CTGF) and cysteine-rich angiogenic inducer 61 (CYR61), in the SQC group (*P* < *0.05,* Fig. 7D). These findings suggest the inactivation of the Hippo-YAP pathway.

## Discussion

Our study provides robust scientific evidence for the treatment of DVC with SQC, while simultaneously revealing multifaceted potential therapeutic mechanisms. This research contributes to the clinical value of SQC in managing diabetes and its vascular complications, laying a solid foundation for future investigations.

Primarily, SQC’s ability to restore aberrant glucose and lipid metabolism suggests a potential means to manage and prevent DVC. Dysregulated lipid metabolism has been associated with calcification and atherosclerosis, connecting lipid-driven atherosclerosis with the calcification cascade (Neels et al. 2023; Natesan and Kim 2021; Gofman and Lindgren 1950). SQC’s potential to address metabolic aspects strengthens its therapeutic potential.

Vascular calcification, a complex process, involves calcium mineral deposition within blood vessel walls (Lee et al. 2020; Villa-Bellosta 2021). Osteogenic differentiation, akin to bone formation, plays a crucial role in this process (Ghosh et al. 2020; Abbasian 2021). By inhibiting calcium deposition and osteogenic differentiation, SQC can interrupt VSMCs phenotypic transition, a critical factor in calcification initiation and progression. Moreover, SQC’s ability to suppress apoptosis is pivotal in preventing aortic complications in diabetes. The observed suppression of apoptosis suggests a protective effect of SQC against cell death in the diabetic aorta, opening doors to novel treatment strategies (Han et al. 2020; Zhao et al. 2020).

Modulation of tissue inflammation responses and ECM remodeling are closely linked to DVC development (Cai et al. 2023). Our findings demonstrate that SQC influences ECM remodeling, which is implicated in vascular calcification (Shantsila and Lip 2009; Lee et al. 2021). Chronic inflammation within blood vessel walls triggers the release of pro-inflammatory cytokines, activating molecular pathways that induce cellular changes conducive to calcification (Cai et al. 2023; Bessueille and Magne 2015). By attenuating inflammatory processes, SQC creates an environment less conducive to calcification.

The Hippo-YAP pathway, vital in physiological processes, has been linked to vascular disorders (Zhou and Zhao 2018). It involves protein interactions and phosphorylation events regulating YAP activity, influencing cell behavior and pathological changes in vascular diseases (Wang et al. 2018b; He et al. 2018; Ma et al. 2019). Multiple factors, including mechanical stretch, inflammatory signals, and changes in extracellular matrix composition, can trigger YAP activation (Zheng et al. 2022). Moreover, the Hippo-YAP pathway has been implicated in arterial calcification (Ma et al. 2022; Shi et al. 2022). SQC’s modulation of this pathway indicates its potential role in mediating therapeutic effects.

This experiment represents an initial, comprehensive, and systematic exploration of SQC’s therapeutic effects on DVC at the animal level, revealing potential mechanisms of action. However, each potential mechanism necessitates rigorous experiments for further validation. Furthermore, in future research, if we intend to extrapolate the dosages administered in the rat model to human dosages, it is imperative to consider fundamental factors such as body weight and metabolic rates. Simultaneously, the potential synergy between SQC and conventional antidiabetic therapies holds substantial promise. However, all of these aspects will require the establishment of additional related studies for further confirmation.

## Conclusion

This study represents a significant step in unraveling the potential of SQC as a therapeutic agent in the context of DVC. The multifaceted mechanisms uncovered in our research underscore the promise of SQC in mitigating this severe diabetic complication. The restoration of aberrant glucose and lipid metabolism, the inhibition of VSMC phenotypic transformation, and the suppression of cell apoptosis point to SQC’s ability to address fundamental processes underlying DVC. Moreover, the modulation of tissue inflammation responses, the remodeling of the ECM, and the activation of the Hippo-YAP signaling pathway provide insights into the molecular complexity of this condition and potential targets for therapeutic intervention.

### Supplementary Information

**Additional file 1.** The Supplementary methods: including section 1 to section 7.

**Additional file 2.** The Supplementary Table 1: the primer sequences of qRT-PCR.

**Additional file 3.** The Supplementary Table 2: the DEmRNAs between model group and control group.

**Additional file 4.** The Supplementary Table 3: the DEmRNAs between SQC group and model group.

**Additional file 5.** The Supplementary Table 4: the top 20 GO terms.

**Additional file 6.** The Supplementary Table 5: the top 20 KEGG pathways.

**Additional file 7.** The Supplementary Figure 1: the level of fasting glucose and body weight.

**Additional file 8.** The Supplementary Figure 2: the serum biochemical marker analysis and H&E staining.

**Additional file 9.** The Supplementary Figure 3: the Hippo signal pathway.

## Data Availability

All data needed to evaluate the conclusions in the paper are present in the paper or the Additional file 1.

## Abbreviations

SQC: Shenqi Compound
DVC: Diabetic vascular calcification
GK: Goto Kakizaki
VSMCs: Vascular smooth muscle cells
ECM: Extracellular matrix
EVG: Verhoeff’s Van Gieson
AST: Aspartate transaminase
ALT: Alanine transaminase
CKMB: Creatine kinase MB
ALP: Alkaline phosphatase
AGEs: Advanced glycosylation end products
MDA: Malondialdehyde
ROS: Reactive oxygen species
TC: Total cholesterol
TG: Triglyceride
Ox-LDL: Oxidize low-density lipoprotein
IL-6: Interleukin 6
IL-4: Interleukin 4
TNF-α: Tumor necrosis factor-α
TGF-β: Transforming growth factor-β
NF-KB: Nuclear Factor Kappa Beta
IL-1β: Interleukin-1β
COX-2: Cyclooxygenase-2
COL1A1: Collagen type I alpha 1
COL1A2: Collagen type I alpha 2
ELN: Elastin
MMP: Matrix metalloproteinase
ACAN: Aggrecan
VCAN: Versican
CTGF: Connective tissue growth factor
CYR61: Cysteine-rich angiogenic inducer 61
