# Supplementary material for: Efficacy and mechanism of Shenqi Compound in inhibiting diabetic vascular calcification
Source: Mol Med. 2023 Dec 13;29:168. doi: 10.1186/s10020-023-00767-7 (PMC10720156; doi:10.1186/s10020-023-00767-7)
Supplement: Supplementary file 1 — Additional file 1. The Supplementary methods: including section 1 to section 7. [file 10020_2023_767_MOESM1_ESM.docx]

### Section 1. Alizarin Red S and Von Kossa staining

1. **Alizarin Red S Staining:**

Dehydration: Dehydrate paraffin-embedded tissue sections using a graded series of alcohol solutions.

Alizarin Red S Solution: the dehydrated sections involved incubation in a 2% solution at pH 4.2 for 30 minutes, allowing calcium deposits to bind with the dye.

Differentiation: Wash the sections with with 1% sodium hydroxide for 5 minutes to remove excess dye.

Dehydration: Dehydrate the sections again using alcohol.

Clearing: Clear the sections with a suitable clearing agent.

Mounting: Mount the sections on glass slides with coverslips.

1. **Von Kossa Staining:**

Deparaffinization and Rehydration: Remove paraffin from tissue sections and rehydrate them using a graded series of alcohol solutions.

Silver Nitrate Solution: Incubate the rehydrated sections with silver nitrate solution and expose them to 1% silver nitrate under ultraviolet light for 1 hour, then treated with a 5% sodium thiosulfate solution for 5 minutes.

Counterstaining: Wash the sections and counterstain them with a suitable dye.

Dehydration: Dehydrate the sections using alcohol.

Clearing: Clear the sections with an appropriate clearing agent.

Mounting: Mount the sections on glass slides with coverslips.

These staining techniques facilitate the visualization of calcium deposits in the tissue sections. The stained sections are then prepared for examination under a light microscope, enabling the observation of calcium-rich areas with distinct staining patterns. Quantitative analysis was performed using ImageJ analysis software.

### Section 2. Masson and Verhoeff's Van Gieson (EVG) staining

### Tissue Preparation: Obtain formalin-fixed arterial tissue sections. Deparaffinize and rehydrate tissue sections through a graded alcohol series. Rinse sections with distilled water.

### Weigert's Iron Hematoxylin Staining: Immerse tissue sections in Weigert's iron hematoxylin solution for 5-10 minutes. Rinse thoroughly with distilled water.

### Differentiation: Place the sections in acid alcohol (1% hydrochloric acid in 70% ethanol) until collagen is the only tissue component staining blue. Rinse sections with distilled water.

### Verhoeff's Solution: Cover the sections with Verhoeff's solution and incubate for 30 minutes. Rinse in 2 changes of 95% ethanol.

### Counterstaining: Counterstain sections with Van Gieson's solution for 5-10 minutes. Rinse with distilled water.

### Dehydration and Mounting: Dehydrate sections through a graded alcohol series. Clear sections in xylene or xylene substitute. Mount with a coverslip using a suitable mounting medium.

### Microscopy: Examine the stained sections under a light microscope. Capture images as needed for analysis.

### **Section 3: Preparation of Vascular Smooth Muscle Cell Suspensions in Arterial Tissue**

### Isolating VSMCs from arterial tissue is a crucial step to investigate their role in arterial calcification and the effects of SQC treatment. Here, we outline the detailed procedure for the preparation of VSMC suspensions from arterial tissue:

### Tissue Harvesting: Arterial tissue, typically from the aorta, was collected from the sacrificed rats. The harvested tissue was placed in a sterile dish containing cold phosphate-buffered saline (PBS) to maintain cell viability.

### Removal of Adventitia: The arterial tissue was carefully dissected to remove the adventitia, leaving behind the tunica media, which primarily consists of VSMCs. This step was performed under a dissecting microscope to ensure precision.

### Minced Tissue: The tunica media was minced into small pieces using sterile scissors and forceps. Care was taken to keep the tissue moist with cold PBS to prevent tissue desiccation.

### Enzymatic Digestion: The minced arterial tissue was transferred to a sterile conical tube and subjected to enzymatic digestion. An enzyme solution containing collagenase and other appropriate enzymes was prepared following the manufacturer's instructions. The tissue fragments were incubated in the enzyme solution at a controlled temperature for a specified time to facilitate cell dissociation.

### Gentle Agitation: During the enzymatic digestion, the conical tubes were gently agitated using a rotator or shaker to aid in tissue disaggregation and the release of VSMCs from the tissue matrix.

### Filtration: Following enzymatic digestion, the cell suspension was passed through a sterile cell strainer with an appropriate pore size to remove undigested tissue fragments. This step ensured the isolation of VSMCs in suspension.

### Washing and Pelleting: The filtered cell suspension was centrifuged to pellet the VSMCs. The supernatant was carefully removed, and the VSMC pellet was resuspended in fresh culture medium suitable for subsequent cell culture or analyses.

### Viability and Cell Count: The viability and concentration of isolated VSMCs were determined using a hemocytometer or an automated cell counter. Viability was assessed using common dyes such as Trypan Blue or propidium iodide.

### Final Suspension: The VSMC suspension was adjusted to the desired concentration with culture medium or appropriate buffer as needed for subsequent experiments.

### **Section 4: Flow Cytometry Assessment of Apoptosis in VSMCs**

### Preparation of VSMC Suspension: Retrieve the VSMC suspension obtained as previously described and ensure it is properly resuspended in culture medium to achieve the desired concentration.

### Annexin V-FITC and Propidium Iodide Staining:

### Centrifuge the VSMC suspension for 5 minutes at 300 × g to pellet the cells.

### Discard the supernatant, and carefully resuspend the VSMC pellet in cold, sterile PBS.

### Centrifuge the cells again for 5 minutes at 300 × g and remove the PBS.

### Resuspend the VSMC pellet in binding buffer at a concentration of approximately 1 × 10^6 cells/ml.

### For each sample, transfer 100 μl (approximately 1 × 10^5 cells) of the VSMC suspension to a flow cytometry tube.

### Add 5 μl of Annexin V-FITC to each sample and gently mix. Incubate for 15 minutes at room temperature in the dark.

### After incubation, add 10 μl of PI solution to each sample, mix gently, and incubate for an additional 5 minutes in the dark.

### Flow Cytometry Analysis:

### a. Following staining, add 400 μl of binding buffer to each sample to bring the volume to 500 μl.

### b. Analyze the samples using a flow cytometer. Ensure proper instrument settings, including compensation and laser configuration.

### c. Use appropriate gating to distinguish viable cells (Annexin V-FITC and PI negative), early apoptotic cells (Annexin V-FITC positive and PI negative), late apoptotic or necrotic cells (Annexin V-FITC and PI positive), and other populations of interest.

### Data Collection and Analysis:

### Collect data from at least 10,000 events per sample.

### Analyze the data using flow cytometry software, and calculate the percentage of cells in each population based on the Annexin V-FITC and PI staining patterns.

### **Section 5: Safety Evaluation of SQC in Rats**

A total of 32 Wistar rats were randomly divided into four groups: the Normal Control (NC) group, Low Dose of SQC (SQC-L) group, Medium Dose of SQC (SQC-M) group, and High Dose of SQC (SQC-H) group, with 8 rats in each group. The NC group was administered sterile distilled water by gavage, while the other groups received SQC working solutions (prepared with sterile distilled water at low (7.2 g/kg/day), medium (14.4 g/kg/day), and high (28.8 g/kg/day) doses) by gavage for a total of 7 days. After the gavage period, the rats were sacrificed. Blood samples were collected from the abdominal aorta for subsequent biochemical analysis. Vital organs, including the heart, liver, spleen, lungs, and kidneys, were harvested for subsequent H&E pathological staining.

### **Section 6. Serum biochemistry**

Blood samples were collected and subsequently centrifuged at 3,000 × g for 15 minutes at 4°C, followed by the biochemical tests, which were performed using the Cobas8000 utomatic analyzer (Roche).

### **Section 7. H&E staining and histopathology**

Following treatment, thoracic aortas, heart, liver, kidney, and lungs were dissected, fixed in 4% paraformaldehyde, and embedded in paraffin for thin sectioning (5-7μm). H&E staining involved deparaffinization, rehydration, and nuclear-cytoplasmic visualization. A blinded pathologist assessed structural integrity, vascular wall architecture, cell morphology, and histopathological changes under a light microscope, systematically examining multiple sections from each group. Findings were documented with captured images.
