# Supplementary figures and images for: Efficacy and mechanism of Shenqi Compound in inhibiting diabetic vascular calcification
Source: Mol Med. 2023 Dec 13;29:168. doi: 10.1186/s10020-023-00767-7 (PMC10720156; doi:10.1186/s10020-023-00767-7)

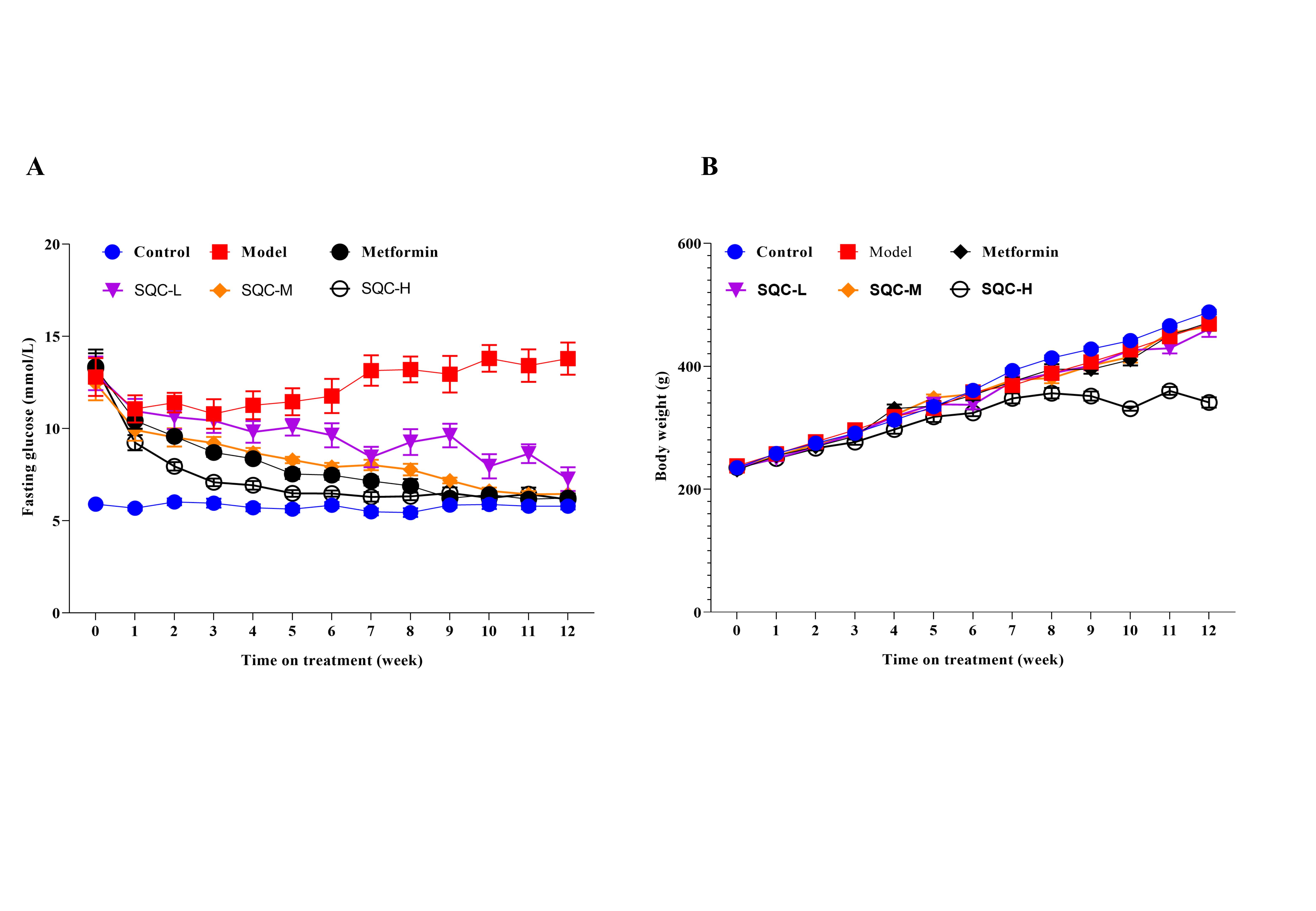

Supplement: Supplementary file 7 — Additional file 7. The Supplementary Figure 1: the level of fasting glucose and body weight. [file 10020_2023_767_MOESM7_ESM.tif]

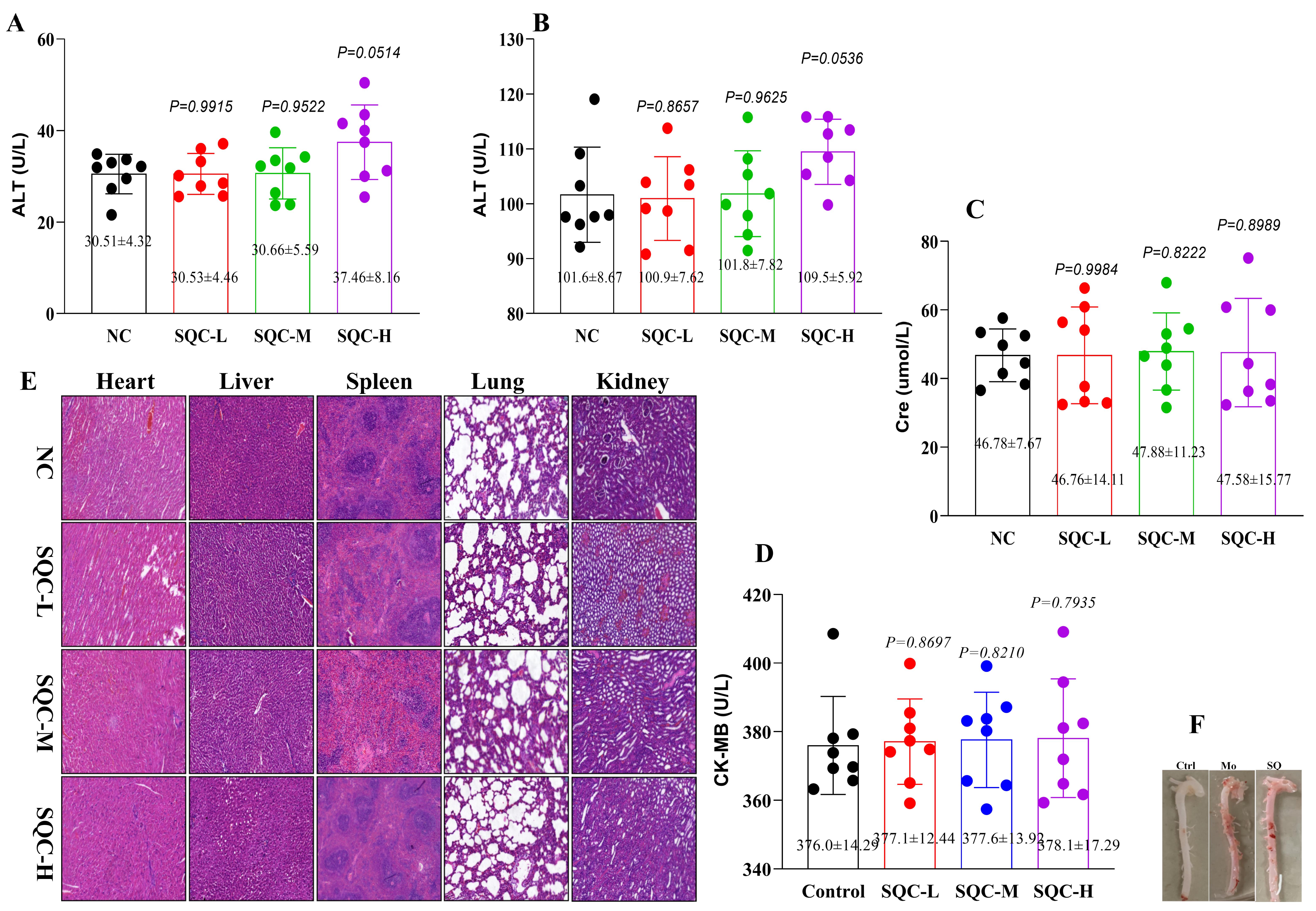

Supplement: Supplementary file 8 — Additional file 8. The Supplementary Figure 2: the serum biochemical marker analysis and H&E staining. [file 10020_2023_767_MOESM8_ESM.tif]

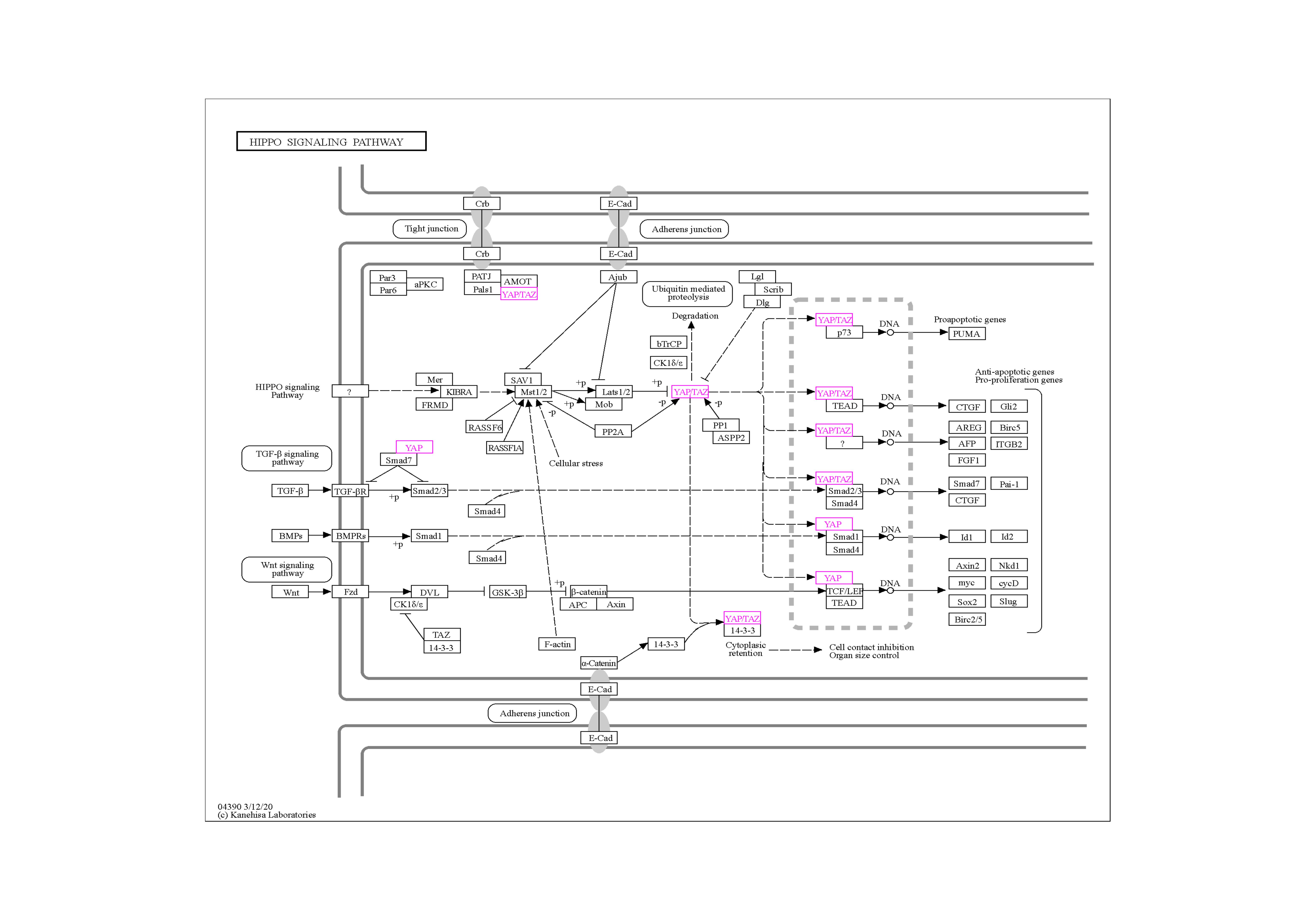

Supplement: Supplementary file 9 — Additional file 9. The Supplementary Figure 3: the Hippo signal pathway. [file 10020_2023_767_MOESM9_ESM.tif]
